# Combined Transforaminal Lumbar Interbody Fusion and Smiley Face Rod Technique Using Dual-Headed Pedicle Screws for L4 Isthmic Spondylolisthesis and L5 Spondylolysis

**DOI:** 10.7759/cureus.60756

**Published:** 2024-05-21

**Authors:** Masaki Tatsumura, Shunsuke Ikezawa, Shun Okuwaki, Hisanori Gamada, Toru Funayama

**Affiliations:** 1 Department of Orthopaedic Surgery and Sports Medicine, Tsukuba University Hospital Mito Clinical Education and Training Center/Mito Kyodo General Hospital, Mito, JPN; 2 Department of Orthopaedic Surgery, University of Tsukuba, Tsukuba, JPN

**Keywords:** pseudarthrosis, adjacent segmental disease, return to sport, preservation of mobile segment, softball, transforaminal lumbar interbody fusion(tlif), spondylolysis, isthmic spondylolisthesis, dual-headed pedicle screw, smiley face rod technique

## Abstract

Spondylolysis with pseudarthrosis may be treated surgically by repairing the spondylolysis using the smiley face rod (SFR) technique. The SFR technique can avoid adjacent segmental disease caused by transforaminal lumbar interbody fusion (TLIF), which is one of the main surgical techniques to treat isthmic lumbar spondylolisthesis.

A 59-year-old woman had been playing softball since she was 12 years old and was a member of a prefectural representative team. She sought treatment because of numbness in her left lower limb and difficulty playing softball. Despite conservative treatment for a year, her symptoms did not improve. Physical examination revealed decreased patellar tendon reflexes and numbness and pain from the front of the thigh to the lower leg without muscle weakness. Imaging showed L4 isthmic spondylolisthesis with Meyerding classification grade 2 anterior slip and L5 spondylolysis with pseudarthrosis. We diagnosed L4 radiculopathy caused by L4/5 foraminal stenosis and L4 isthmic spondylolisthesis with L5 spondylolysis. She underwent surgery combining the TLIF of L4/5 and the SFR technique of L5 using dual-headed pedicle screws that can fix two types of rods with L5 pedicle screws. Three months after surgery, fusion between L4/5 and fusion of the L5 pars cleft were confirmed. She resumed sports, and one year postoperatively, she was able to participate in softball games. Two years postoperatively, she could bat, run, and play defense without adjacent segmental disease.

Two-segment TLIF increases adjacent segmental disease more than single-segment TLIF. Because the L5 spondylolysis had not slipped, we chose the SFR technique to preserve mobility at L5/S1. The dual-headed pedicle screw fastens two-type rods at the head of the pedicle screw, making it a suitable design for this procedure.

## Introduction

Transforaminal lumbar interbody fusion (TLIF) is one of the main surgical techniques to treat isthmic lumbar spondylolisthesis with lower extremity pain [1]. Spondylolysis with pseudarthrosis may be treated surgically by repairing the spondylolysis using the smiley face rod (SFR) technique [2]. We report a case of 4th lumbar (L4) isthmic spondylolisthesis and 5th lumbar (L5) spondylolysis without slip, in which a combination of TLIF and SFR with a dual-headed pedicle screw was performed.

## Case presentation

**Figure 1:**
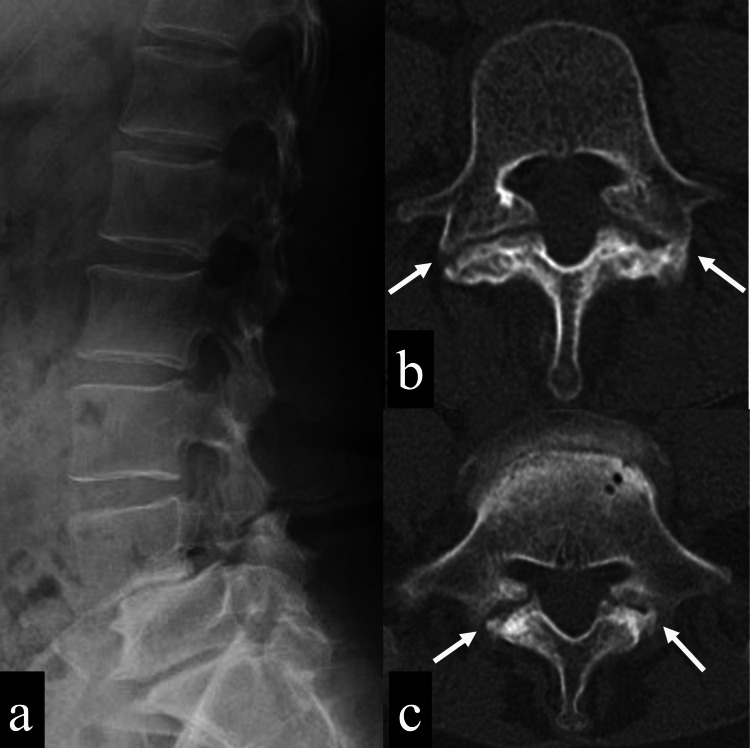
Preoperative imaging 1a: A plain lateral X-ray image of the lumbosacral spine shows L4 isthmic spondylolisthesis with Meyerding classification grade 2 anterior slip and L5 spondylolysis without slip. 1b: A plain CT axial view (L4 level) shows pseudoarthrotic isthmic spondylolisthesis at the L4 (white arrows). 1c: A plain CT axial view (L5 level) shows pseudoarthrotic spondylolysis at the L5 (white arrows).

A 59-year-old woman had been playing softball since she was 12 years old and was a member of a prefectural representative team. She sought treatment because of numbness in her left lower limb and difficulty playing softball. Despite conservative treatment for a year, her symptoms did not improve. Physical examination revealed decreased left patellar tendon reflexes and numbness and pain from the front of the left thigh to the lower leg without muscle weakness. Imaging showed L4 isthmic spondylolisthesis with Meyerding classification grade 2 anterior slip and L5 spondylolysis (Figure 1).

**Figure 2:**
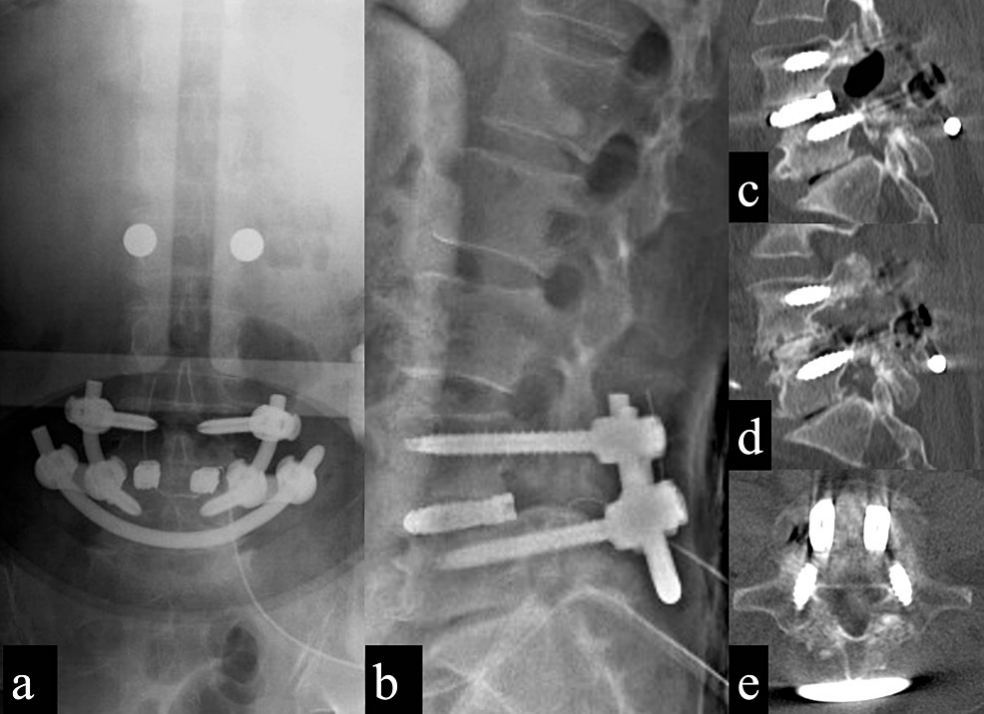
Imaging findings after transforaminal lumbar interbody fusion (TLIF) of L4/5 and smiley face rod (SFR) of L5 2a: A plain AP X-ray image of the lumbosacral spine shows TLIF of L4/5 and SFR of L5 performed using dual-headed pedicle screws. 2b: A plain lateral X-ray image of the lumbosacral spine shows a reduction of L4 anterior slip. 2c: A plain CT sagittal view (right) shows no gap at the L5 pars cleft. 2d: A plain CT sagittal view (left) shows no gap at the L5 pars cleft. 2e: A plain CT axial view shows no gap at the L5 bilateral pars clefts.

We diagnosed L4 radiculopathy caused by L4/5 foraminal stenosis and L4 isthmic spondylolisthesis with L5 spondylolysis. She underwent surgery combining TLIF of L4/5 and SFR of L5 using dual-headed pedicle screws that can fix two types of rods with L5 pedicle screws (Figure 2).

**Figure 3:**
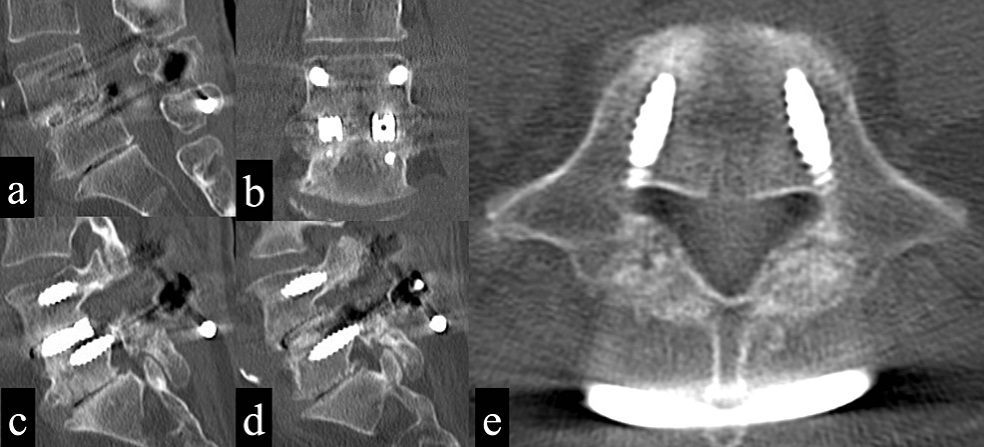
Imaging findings of three months after surgery 3a: A plain CT sagittal view (midline) shows bony bridging between L4/5. 3b: A plain CT coronal view shows bony bridging between L4/5. 3c: A plain CT sagittal view (right) shows bony fusion at the L5 right pars cleft. 3d: A plain CT sagittal view (left) shows bony fusion of the L5 left pars cleft. 3e: A plain CT axial view showing bony fusion at the L5 bilateral pars clefts.

Three months after surgery, a fusion between L4/5 and fusion of the L5 pars cleft was confirmed (Figure 3).

**Figure 4:**
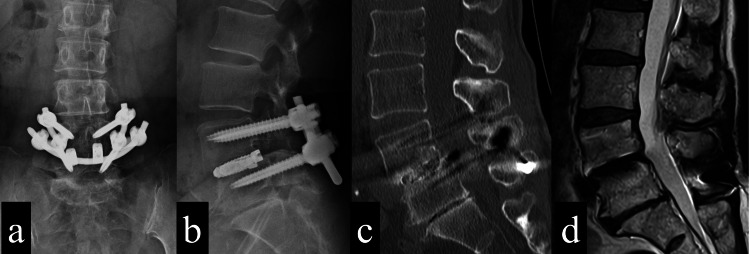
Imaging findings two years after surgery 4a: A plain AP X-ray image of the lumbosacral spine shows no adjacent segmental disorders. 4b: A plain lateral X-ray image of the lumbosacral spine shows no adjacent segmental disorders. 4c: CT sagittal view (midline) shows intervertebral disc height was not changed compared to preoperative height. 4d: MRI sagittal view (midline) shows no canal stenosis.

She resumed sports, and one year postoperatively, she was able to participate in softball games. Two years later, postoperatively, she could bat, run, and play defense. Regarding her physical examination, her decreased patellar tendon reflexes were not changed, but the numbness and pain disappeared, and there was no muscle weakness. Clinical images also showed no implant failure and no adjacent segmental disease (Figure 4).

## Discussion

TLIF between L4 and the first sacral vertebra (S1) is the standard procedure for spondylolysis of L4 and L5. The patient was middle-aged, but her athletic performance level was high, and she wished to return to competition after surgery. As the L4/5 intervertebral foramen stenosis due to L4 isthmic spondylolisthesis required decompression, TLIF was selected for the L4/5 segment. Surgery for L5 spondylolysis was also considered necessary. However, if L5/S1 were also treated with TLIF, the 2-segment TLIF would limit her range of motion [3]. Therefore, because the L5 spondylolysis had not slipped, we chose SFR to preserve the mobile segment at L5/S1.

Two-segment TLIF increases adjacent intervertebral disorders more than single-segment TLIF. We were concerned about adjacent segmental disease because the patient wanted to return to sports. We felt that intervertebral fixation of only L4/5 would prevent degeneration of the L3/4 intervertebral segment more than fixation of L4/5/S.

Recent surgical techniques for lumbar spondylolysis with pseudarthrosis include the hook rod technique [4] or the SFR technique [2]. The beginning of history of the SFR technique was reported as the V-rod technique began by Gillet et al. in 1999 [5]. Later, Ulibarri et al. improved it and reported it as the U-shaped rod technique [6]. Voisin et al. named it the SFR technique [7] and further applied it to its current technique [8].

Crawford et al. reported that up to 74% of lumbar spondylolysis patients develop isthmic spondylolisthesis [9], and one of the advantages of SFR is the reduction capacity of spondylolisthesis up to the Meyerding classification grade 2 anterior slip [10]. However, SFR is not indicated for elderly patients, who often have more than grade 1 slips. In the present case, TLIF was performed because SFR was not indicated for L4. On the other hand, L5 was judged to be an indication of SFR because of minimal slippage.

**Figure 5:**
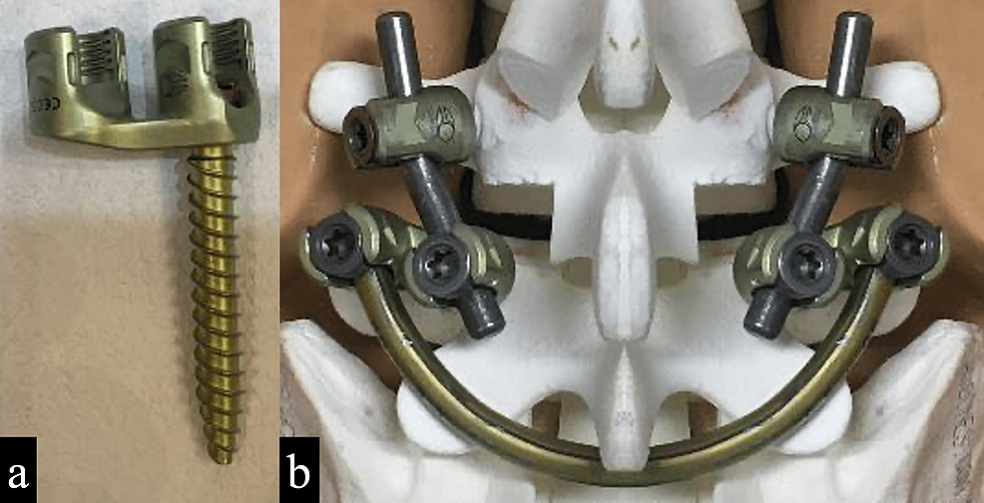
Dual-headed pedicle screw 5a: Dual-headed pedicle screw; there are two screw heads on one pedicle screw. 5b: The schematic in a bone model shows the placement of screws and rods for TLIF and SFR.

As two types of rods in TLIF and SFR are required to insert the L5 pedicle screw, a dual-headed pedicle screw was used to facilitate the combination of TLIF and SFR (Figure 5).

The patient had a bone fusion of the L4/5 vertebral bodies and the L5 pars cleft and was able to return to softball. As mentioned earlier, SFR is often not indicated in older patients due to progressive slippage. Many reports of SFR have been made by young patients. The present case is an approximately 60-year-old case, and the fact that bone fusion was achieved by SFR in older is of great value.

It is not impossible to complete the TLIF of L4/5 and SFR of L5 with a single rod using a conventional screw. However, TLIF requires the application of compression force in the cephalocaudal direction, while the SFR requires the application of compression force in the anteroposterior direction [8]. This screw allows separate compression forces to be applied and avoids the complications of rod bending during surgery.

The dual-headed pedicle screw fastens two rods at the head of the pedicle screw, making it a suitable design for this procedure. However, the caudal side of the TLIF rods should be bent slightly inward because the two rods should be parallel.

## Conclusions

We performed the surgery combining transforaminal lumbar interbody fusion (TLIF) of L4/5 and smiley face rod (SFR) technique of L5 to the case of L4 radiculopathy caused by L4/5 foraminal stenosis and L4 isthmic spondylolisthesis with L5 spondylolysis. She resumed sports and obtained fusion between L4/5 and fusion of the L5 pars cleft were confirmed. The dual-headed pedicle screw is useful for the TLIF of L4/5 and SFR of L5 in cases of L4 isthmic spondylolisthesis and L5 spondylolysis.
